# A Case Report of Stuck Thrombus Aspiration Catheter (Thrombuster) in Left Anterior Descending Artery of A 59‐Year‐Old Patient Undergoing PCI for Revascularization

**DOI:** 10.1002/ccr3.70269

**Published:** 2025-03-05

**Authors:** Alifa Sabir, Muhammad Sohail Chaudhri, Muhammad Azam, Zahid Amin, Basam Azhar, Muhammad Ibrahim, Sameen Mukhtar, Javed Iqbal

**Affiliations:** ^1^ Rawalpindi Institute of Cardiology Rawalpindi Pakistan; ^2^ Rawalpindi Medical University Rawalpindi Pakistan; ^3^ Dow Medical College Karachi Pakistan; ^4^ Nursing Department Communicable Diseases Center Hamad Medical Corporation Doha Qatar

**Keywords:** acute ST‐elevation MI, PCI, percutaneous coronary intervention, percutaneous thrombus aspiration, STEMI, thrombuster

## Abstract

Hardware entrapment during percutaneous coronary intervention is a significant complication necessitating proactive anticipation by interventionists. Here we present a case of a 59‐year‐old man with a history of stent placement in LAD 1 month back, who now presented with stent thrombosis and acute ST‐elevation MI. Percutaneous intervention was done for thrombus aspiration through the previously deployed stent, but the thrombus aspiration device (thrombuster) got stuck in the stent. Despite attempted percutaneous retrieval methods, the device remained inaccessible, leading to emergency surgical device retrieval and simultaneous coronary bypass.

**Summary**

- Complications post interventional procedures, though uncommon, can possibly occur.
- Probable causes include improper handling of the catheters and tortuosity of the coronary vessels.
- If occurred, safe removal should be attempted but when failed prompt surgical removal and revascularization should be ensured.

## 1 Introduction

Stent thrombosis, though uncommon, ranging from 0.5% to 2.2%, can occur as a potential complication after primary PCI, depending on the material of the stent, coronary anatomy, and medication compliance status, resulting in ischemia of the myocardial wall being perfused [1]. Usage of a thrombus aspiration catheter during the percutaneous coronary intervention (PCI) for ST‐elevation myocardial infarction in stent thrombosis yields conflicting outcomes. According to the TOTAL trial, routine thrombus aspiration during PCI for STEMI did not reduce longer‐term clinical outcomes and might be associated with an increase in stroke [2]. However, it dramatically reduces index hospitalization and improves TIMI flow, myocardial blush grade, ST‐segment resolution, and left ventricular systolic function. Nevertheless, complications may arise, and the technique of dealing with them is crucial as no single technique will always be effective. It is worthwhile to be familiar with available techniques and local center availability [3]. We report a case of a stuck thrombus aspiration catheter (thrombuster) retrieved during emergency CABG due to complications of minimally invasive intervention in the catheterization lab.

## 2 Case History

A 59‐year‐old male, diabetic, hypertensive, active smoker, with a history of angioplasty (stent placed in proximal LAD) done for acute MI 1 month back, presented in ER with a pain score ranging from 6 to 7. Pain was associated with sweating and dizziness lasting a few hours before arrival at the emergency department. No signs of heart failure were noted. Blood pressure was 130/70 mmHg, and the pulse rate was 76 bpm. Chest X‐ray did not show signs of pulmonary congestion. Electrocardiography (ECG) showed ST elevations in leads V3 and V4 and T‐wave inversions in all chest leads. Laboratory testing recorded elevated levels of creatine phosphokinase 4295 U/L (normal range 0–190 U/L), Creatine kinase‐MB 331 U/L (normal range 0–25 U/L), and LDH 1046 (Normal range 0–480 U/L). Aspirin and clopidogrel, together with a subcutaneous Factor Xa inhibitor, were started for acute coronary syndrome treatment. The serial ECG noted dynamic changes and persistent chest pain despite medical therapy, guarded for urgent percutaneous coronary intervention (PCI).

## 3 Diagnosis and Management

**FIGURE 1:**
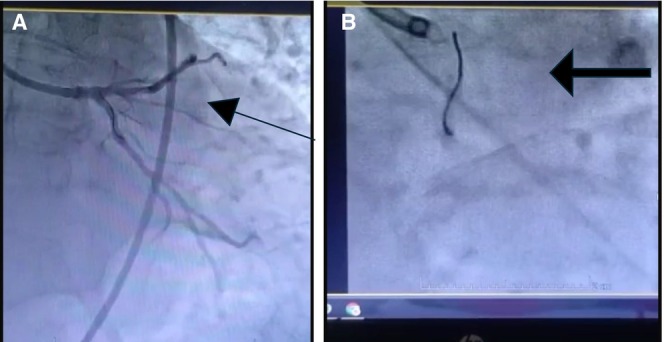
(A) Coronary angiography showing stuck thrombus aspiration catheter (thrombuster) in proximal (left anterior descending) LAD artery previously placed stent. Arrow points towards previously deployed stent with thrombuster crossing the stent. (B) Coronary angiography showing stuck thrombus aspiration catheter (thrombuster) in proximal LAD artery. The arrow points towards the stuck thrombuster.

Trans‐radial coronary angiogram (through the right radial artery) revealed three major coronary arteries with stent thrombosis in the proximal segment of the left anterior descending artery at the stent inlet; the culprit lesion was identified. The left coronary system was engaged with 6F EBU 3.5 as a guide, guiding the catheter through the right radial access with a 6F sheath. The lesion was crossed using Choice Floppy, two PT Graphix guide wires, and the stent was placed with a 2.5 × 15 mm Sphincter balloon and 3 × 15 mm NC Euphora. A thrombuster was used to give intracoronary Aggrastat and Nitro, but distal flow could not be achieved, while removing the thrombuster got engaged in the stent strut (Video 1). A telescope catheter was used, but it did not work. The distal portion of the stent was deformed (Figure 1). The patient became vitally unstable.

**VIDEO 1:** As shown in the video (Click to play), the coronary angiogram reveals a thrombus aspiration catheter (thrombuster) stuck in the proximal LAD artery. The footage highlights the catheter's position, vascular anatomy, and the technical challenges involved in revascularization. Video content can be viewed at https://onlinelibrary.wiley.com/doi/10.1002/ccr3.70269

**FIGURE 2:**
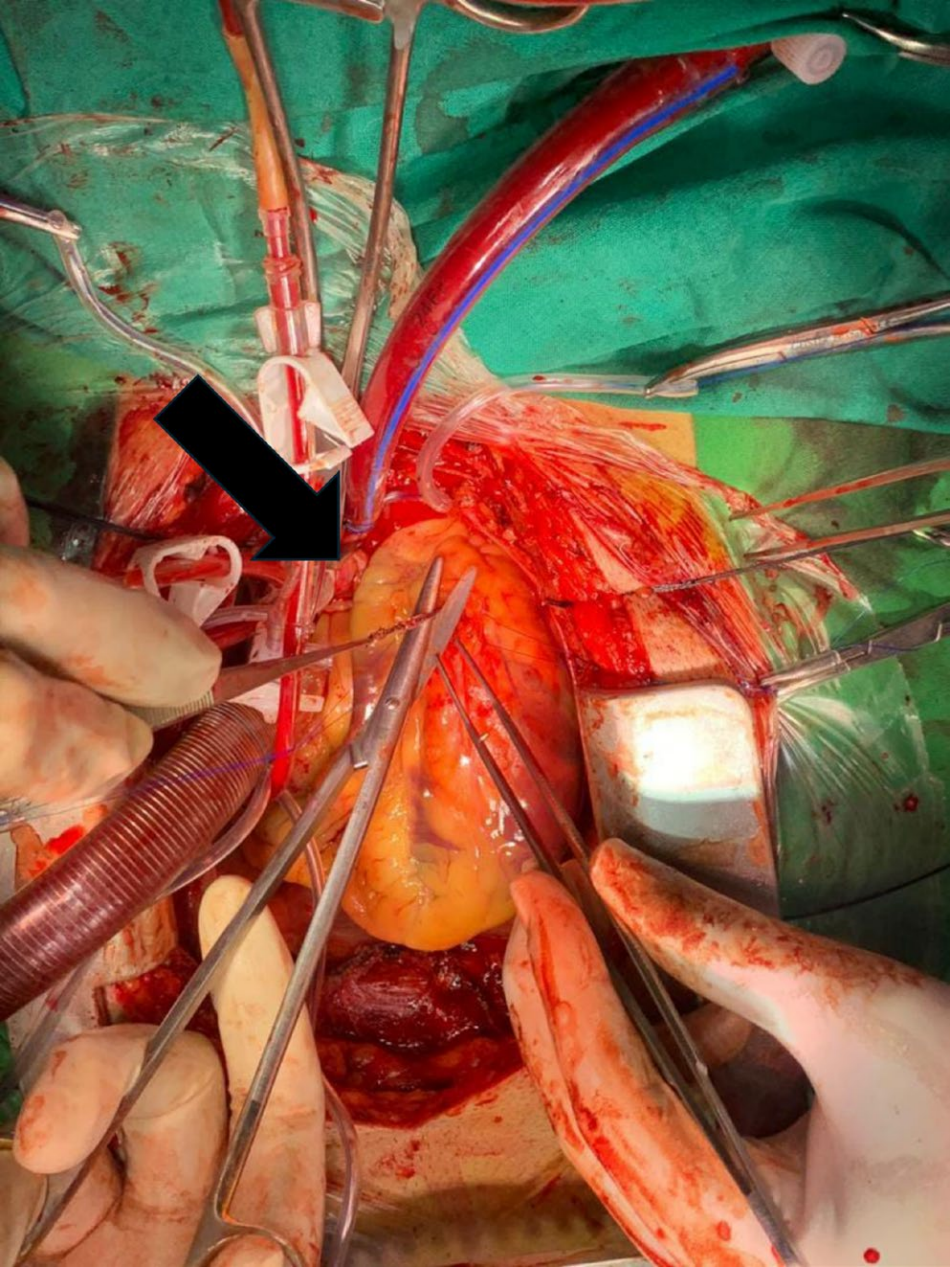
Retrieval of thrombus aspiration catheter (thrombuster) after exposing proximal LAD. The arrow shows thrombuster.

**FIGURE 3:**
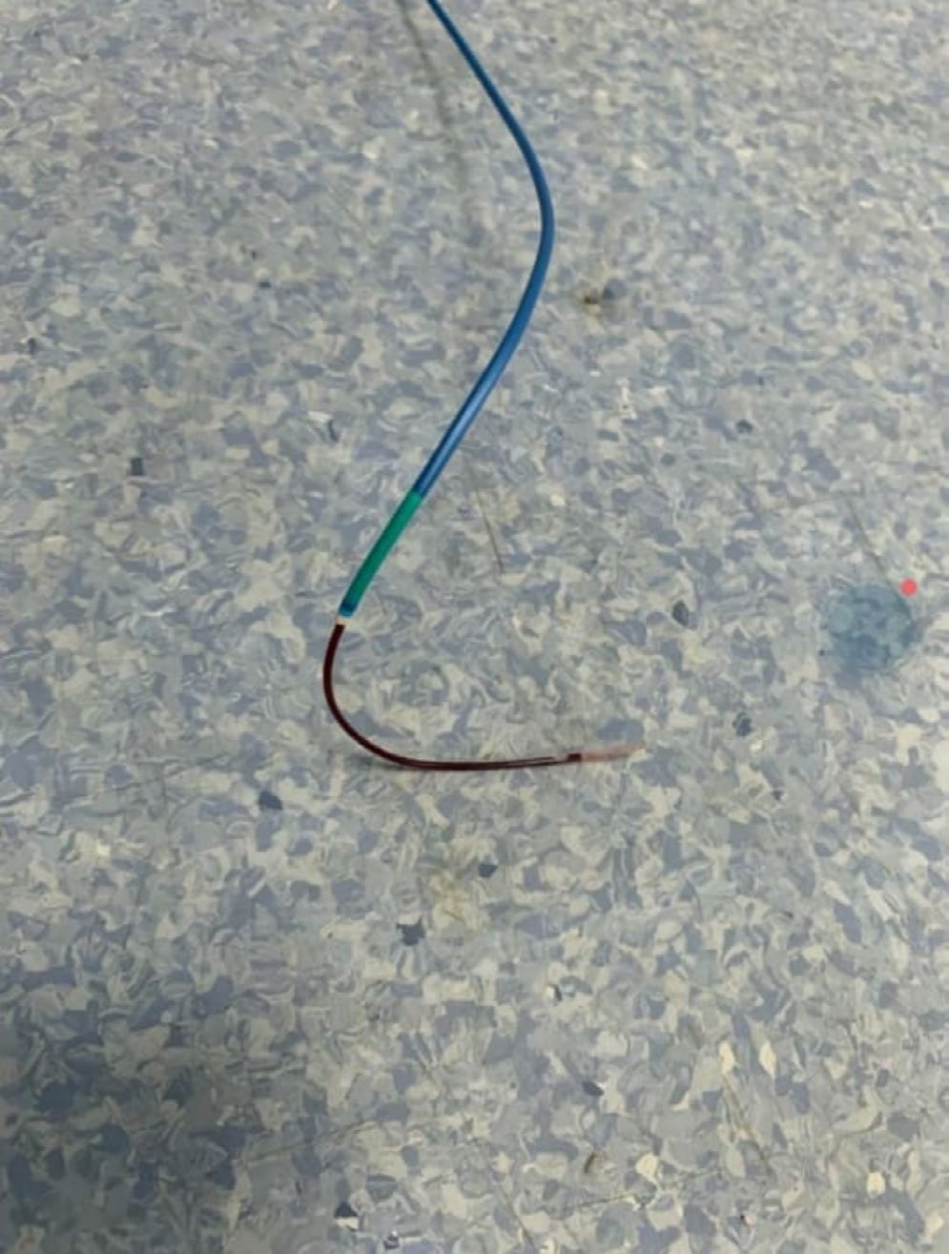
Retrieval of the guide wire through radial artery.

**FIGURE 4:**
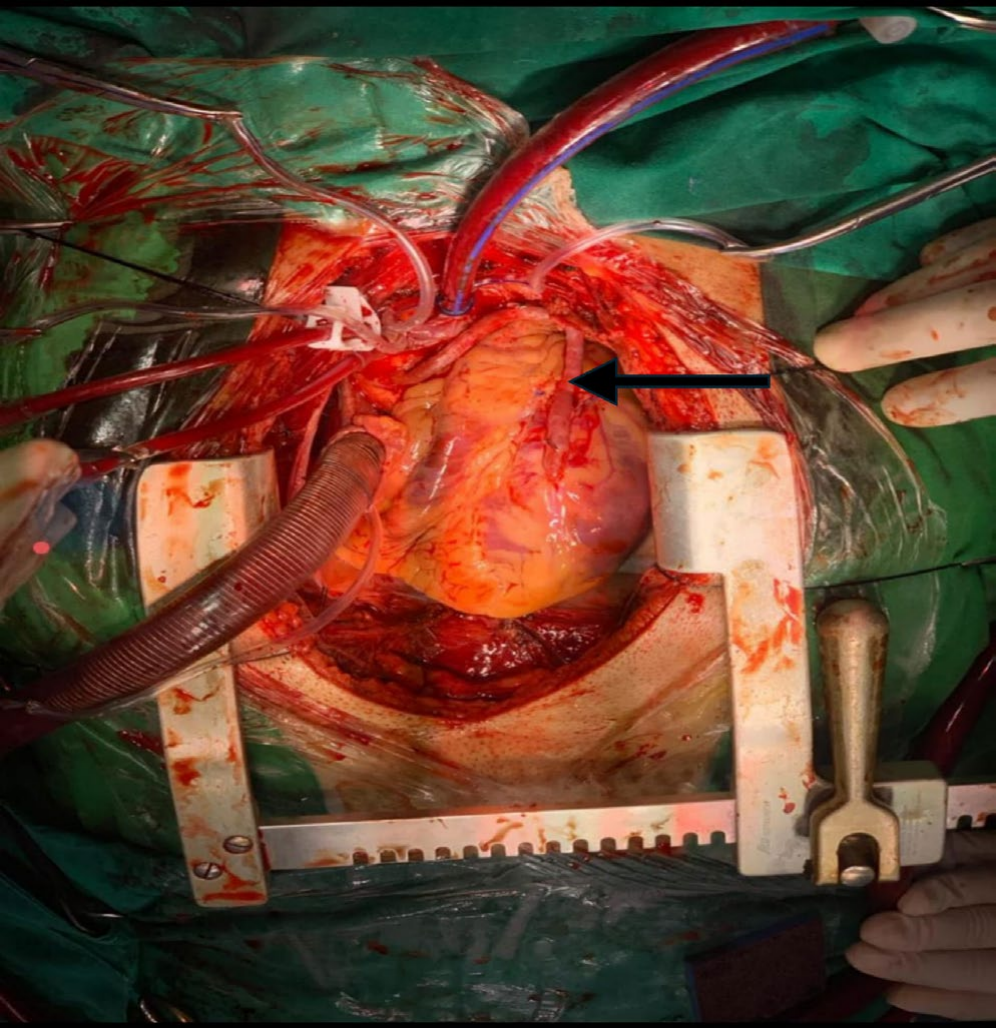
Saphenous vein graft from aorta to proximal LAD.

IABP was placed, and the patient was shifted to the OR for emergency CABG for hardware retrieval and CABG. A median sternotomy was done, and the pericardium was opened. Heparin was given. Aortic and two staged venous cannulations were done. CPB was established. Cross clamp was applied. Antegrade cardioplegia was given. Heart stopped. LAD was opened. The impacted thrombuster catheter was freed from the stent and retrieved along with the previously deployed stent. (Figure 2) The rest of the catheter was pulled out through the radial artery. (Figure 3) Endarterectomy of LAD and great saphenous vein patchplasty was done over the LAD. The top end of the vein graft was then anastomosed to the aorta. (Figure 4) CPB was weaned off and de‐cannulation was done. Protamine was given, and the chest was closed.

## 4 Results

The patient was shifted to ICU, where he was extubated smoothly. IABP was weaned off on the 2nd POD. The patient had an uneventful recovery and was discharged on the 5th POD.

## 5 Discussion

In this case, we are dealing with stent thrombosis in the Left anterior descending coronary artery in a 59‐year‐old male, diabetic, hypertensive, active smoker, with a history of angioplasty (stent placed in proximal LAD) done for acute MI 1 month back [4]. We experienced a stuck thrombus aspiration catheter (thrombuster), which was a previously unreported complication. An individualized approach to managing patients with a considerable thrombus burden in acute coronary syndrome still demands the use of a thrombus aspiration catheter despite unfavorable outcomes for the routine use of thrombus aspiration (class III, A recommendation) [5]. The TOTAL trial concluded that routine manual thrombectomy did not reduce the risk of cardiovascular death, recurrent MI, cardiogenic shock, or NYHA class IV heart failure within 180 days but was associated with an increased rate of stroke within 30 days, according to the trial [6] According to the TASTE trial, routine thrombus aspiration before PCI did not reduce 30‐day mortality; furthermore, there were no significant differences between the two groups (manual thrombus aspiration before PCI and PCI only) concerning the rate of stroke or neurologic complications at the time of discharge. Thrombuster was designated for improved flexibility and smooth insertion into a vessel with a hydrophilic coating over the first 30 cm from the tip of the catheter. Improved aspiration was achieved by lowering flow resistance with a large and circular aspiration lumen. As only a few case reports are recorded, the complication rate of thrombuster is unknown. Cagdas Akgullu et al. reported a trapped thrombus aspiration catheter in the coronary artery due to a rupture of its main shaft and twisting over the wire, which was successfully managed to be pulled out with the whole system [7]. The other case report noted a thrombus in the left main as one of the complications, and the other reported an inadvertent coronary endarterectomy with a thrombuster III GR catheter [8]. The increasing number of percutaneous coronary interventions over the last few years has led to an increasing number of complications, including fractures and retained hardware. The incidence of entrapment and fracture of guidewire is approximately 0.2% [9]. 48 case reports with a total of 67 patients experienced guidewire entrapment—41.8% underwent percutaneous extraction, 43.3% were managed by surgery, and 14.9% received conservative therapy. Several percutaneous techniques are used, such as stenting against the vessel wall, snare loop, double or triple wire technique, bioptome, tornus micro‐catheter, deep‐guide catheter wedging with balloon inflation, and pigtail catheter [9]. According to one of the case reports on the prevalence and outcome of fractured hardware, 12 patients out of 5400 PTCA procedures experienced different types of retained components, such as guidewire fragments, balloon catheters, and guide catheters. Follow‐up on patients with retained wire had no clinical sequelae during follow‐up ranging from 6 to 60 months, suggesting that the decision to attempt retrieval is based on an individualized approach. Not all patients with retained hardware need to proceed with CABG, especially if the item was retained in an occluded or distal artery [10]. Using the mother–child technique, a fractional flow reserve (FFR) wire tip was successfully retrieved with an angiographic catheter (Slip—Cath). Successful retrieval of broken export catheters by using balloon inflation was reported previously [11].

Possible causes of most retained hardware include excessive torquing, forceful withdrawal of the catheter, inappropriate catheter handling, reuse, manufacturing flaws, and the inadvertent passage of a large catheter through smaller‐sized access sheaths, polymer aging, or a combination of factors. In this case, we analyzed that the thrombuster catheter was stuck, most likely due to irregularity of the coronary vasculature anatomy and properties of the in situ stent, which impeded the extraction of the catheter and obstructed the flow through the affected vessel consequently. The learning points from this complication are as follows: Firstly, checking the aspiration catheter before introducing it into the guide catheter for possible manufacturer defects is essential. Secondly, it is crucial to have a coaxial guiding catheter engagement, a good support/extra support guide wire, gentle maneuvering of the stent retrieval catheter under good imaging fluoroscopy, and inserting the IABP as early as possible to ensure adequate perfusion of other coronary arteries and to reduce the workload on the heart. In the meantime, a surgical team should be gathered to remove the stuck portion to prevent any adverse event surgically, and revascularization of all the occluded vessels suitable for CABG should be done during the same procedure. The need for an efficient surgical team near the cardiology unit is significant as, though rarely, if a condition arises where time is the key to salvaging the myocardium and saving a life, a prompt surgical response could be initiated to avoid adverse outcomes.

## Author Contributions

**Alifa Sabir:** conceptualization, writing – original draft. **Muhammad Sohail Chaudhri:** writing – original draft. **Muhammad Azam:** writing – original draft. **Zahid Amin:** writing – original draft. **Basam Azhar:** writing – original draft. **Muhammad Ibrahim:** writing – original draft. **Sameen Mukhtar:** writing – review and editing. **Javed Iqbal:** writing – review and editing.

## Consent

Written informed consent was obtained from the patient. The patient provided informed consent, understanding that this information might become publicly accessible.

## Conflicts of Interest

The authors declare no conflicts of interest.

## Data Availability

Data will be available on request from the corresponding author.
